# Flotillin-1 enhances radioresistance through reducing radiation-induced DNA damage and promoting immune escape via STING signaling pathway in non-small cell lung cancer

**DOI:** 10.1080/15384047.2023.2203332

**Published:** 2023-05-02

**Authors:** Yingying Wang, Lu Meng, Shuyan Meng, Litang Huang, Shilan Luo, Xiaoting Wu, Xiaomei Gong

**Affiliations:** a Department of Radiation Oncology, Shanghai Pulmonary Hospital, Tongji University School of Medicine, Shanghai, China; b Department of Oncology, Shanghai Pulmonary Hospital, Tongji University School of Medicine, Shanghai, China

**Keywords:** Flotillin-1, radiation resistance, stimulator of interferon genes, programmed death-ligand 1, immune escape

## Abstract

Radiation resistance results in the recurrence and metastasis of non-small cell lung cancer (NSCLC) after radiotherapy. A major cause of radiation resistance is subversion of immune surveillance and clearance. Although our previous research has demonstrated that programmed death-ligand 1 (PD-L1) is responsible for radiation resistance in NSCLC, PD-L1 alone was not a reliable predictor of radiotherapy efficacy. For further exploration of the predictors of radiotherapy efficacy, which could add accuracy to the single biomarker – PD-L1, immunoprecipitation followed by mass spectrometry assay was performed to identify proteins that interact with PD-L1, and flotillin-1 (FLOT1) was detected as a candidate. However, the role of FLOT1 in radiation resistance in NSCLC is largely unknown. Here, we defined FLOT1 as a positive regulator of PD-L1 at the cell level, and the expression of PD-L1 was reduced following FLOT1 depletion. Furthermore, we found that the knockdown of FLOT1 impeded radiation-mediated cell migration and epithelial–mesenchymal transition process. Moreover, FLOT1 depletion enhanced radiation-induced DNA damage, thereby increasing the radiation lethality for NSCLC cells and promoting radiation-mediated tumor regression in animal models and patients with NSCLC. Furthermore, FLOT1 depletion-boosted DNA damage activated STING signaling pathway and promoted the production of CCL5 and CXCL10 that can drive CD8+ T lymphocytes chemotaxis, thereby reprogramming tumor immune microenvironment and triggering the antitumor immune response. Indeed, FLOT1 expression correlated with infiltration of immune cells in NSCLC tumor tissue samples. Taken together, our findings reported an unexplored role of FLOT1 in radiotherapy and also provided an evidence base for FLOT1 as a promising biomarker to predict the response to radiotherapy and a potential therapeutic target for enhancing radiotherapy effects.

## Introduction

Lung cancer remains the leading cause of cancer mortality worldwide, accounting for 18.0% of the total cancer deaths.^1^ Almost 85% of all lung cancer cases are categorized as NSCLC.^2^ Radiotherapy plays an important role in the treatment of NSCLC patients with different stages, whereas the efficacy is restricted due to inherent or acquired radioresistance mediated by cell-intrinsic factors and extracellular microenvironment, particularly the immune microenvironment.^3^

PD-1 expressed by immune cells can interact with PD-L1 expressed by tumor cells, which inhibits anti-tumor immunity and contributes to immune evasion. The use of inhibitors of programmed cell death protein 1 (PD-1) and programmed cell death ligand 1 (PD-L1) has become the standard therapy for the first- or second-line treatment of NSCLC, despite that the efficacy of immunotherapy with chemotherapy was not improved compared with chemotherapy alone in some studies.^4^ Moreover, the level of PD-L1 is currently utilized as a biomarker to evaluate the efficacy of anti-PD-1/PD-L1 therapy in NSCLC in the clinic.^5^ Niki et al. found that the level of PD-1/PD-L1 co-location was significantly correlated with the outcomes of NSCLC treated with anti PD-1/PD-L1 therapies.^6^ Our previous study discovered that NSCLC cells surviving from radiotherapy showed higher expression of PD-L1 to resist radiation through stimulating cell migration, facilitating the epithelial–mesenchymal transition (EMT), suppressing apoptosis, and promoting immune escape. Moreover, NSCLC patients with positive PD-L1 expression had a poorer prognosis than those with negative PD-L1 expression following radiotherapy.^7,8^ However, in the PACIFIC study, patients with PD-L1 expression levels lower than 25% obtain similar survival benefits to patients with higher PD-L1 levels, which demonstrated that PD-L1 alone was not a reliable predictor of radiotherapy efficacy. To further explore the novel predictors to complement the prediction effect of PD-L1, immunoprecipitation followed by mass spectrometry (IP-MS) assay was performed to identify the potential proteins that bound and interact with PD-L1. Based on the MS analysis, a total of 29 proteins were considered as candidates (Supplementary material 1). We eventually focused our research on FLOT1, a marker of lipid raft. The major reasons include the following two aspects: on the one hand, FLOT1 performs a unique function in maintaining protein stability and modulating protein expression levels through mediating internalization, recycling, lysosomal targeting, and redistribution of protein molecules.^9–11^ On the other hand, evidence increasingly supports that FLOT1 correlates with tumor malignancies, such as cell proliferation and tumor growth,^12,13^ cell invasion and metastasis,^14^ and poor prognosis and low survival rate.^15,16^ However, there is a lack of evidence that FLOT1 is involved in radioresistance in NSCLC.

Radiotherapy induces the activation of both innate and adaptive immune responses against tumors through stimulating the cGAS/STING signaling pathway, which is attributed to radiation-induced DNA double-strand breaks (DSBs).^17^ Mitotic progression following DSBs leads to the formation of micronuclei containing DNA.^18^ Breakdown of micronuclear envelope exposes DNA to cytosolic DNA sensor cGAS.^19^ cGAS/STING pathway-mediated CCL5, CXCL10, and type I interferon (IFN) contribute to the chemotaxis and activation of CD8+ T lymphocytes, respectively,^20^ which reprograms tumor immune microenvironment and enhances antitumor immune response. Here, we found that the depletion of FLOT1 boosted radiation-induced DNA damage, hence it is worth exploring whether FLOT1 regulates the STING signaling pathway and the transition from an immunogenic tumor to a non-immunogenic tumor in NSCLC.

This study mainly explored the relationship between FLOT1 expression and radioresistance in NSCLC and then investigated the underlying molecular mechanisms. We finally demonstrated that FLOT1 might enhance radioresistance through facilitating EMT process, suppressing radiation-induced DNA damage, and reprogramming the tumor immune microenvironment via STING signaling pathways in NSCLC. Collectively, these results indicated that FLOT1 was a potential biomarker to predict the efficacy of radiotherapy in clinics. Furthermore, our findings provided a strong rationale for eliminating the radiation resistance by targeting FLOT1 expression.

## Results

### FLOT1 expression was increased in radioresistant NSCLC cells

**Figure 1.**
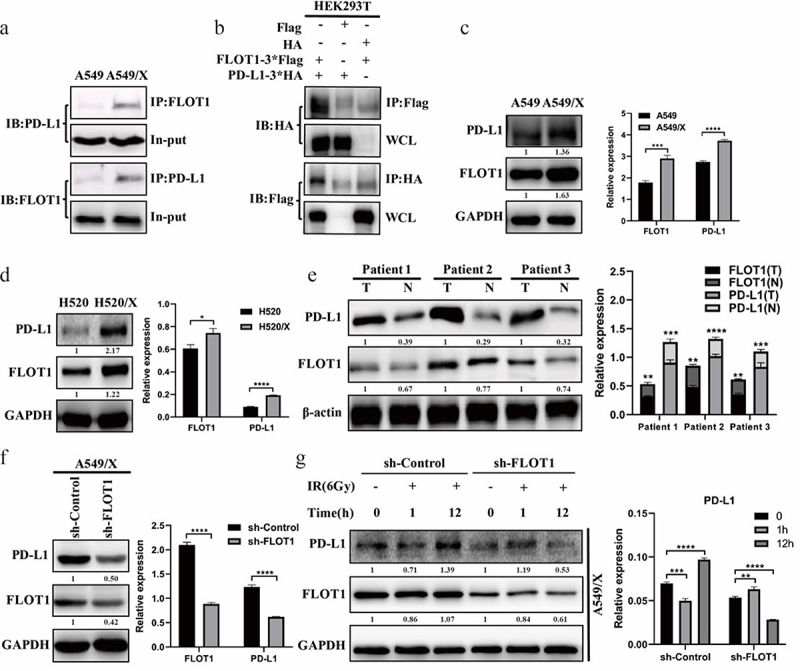
The expression of PD-L1 was reduced following FLOT1 depletion. (a) Endogenous Co-IP reactions of FLOT1 with PD-L1 in A549 and A549/X cells. (b) Immunoblot results of the whole cell lysates (WCL) and anti-Flag/anti-HA immunoprecipitates from HEK293T cells transfected with Flag-FLOT1 plus HA-PD-L1, Flag-FLOT1 plus HA-vector, and HA-PD-L1 plus Flag-vector, respectively. (c and d) Western blotting analysis of FLOT1 and PD-L1 expression in parental cells and radioresistant cells (A549 vs. A549/X; H520 vs. H520/X). (e) Western blotting analysis of FLOT1 and PD-L1 expression in three paired primary NSCLC tissues (T) and matched adjacent nontumor tissues (N) from the same patient. (f) Western blotting analysis of FLOT1 and PD-L1 expression in A549/X cells transduced with shRNA targeting FLOT1 or control shRNA. (g) Western blotting analysis of FLOT1 and PD-L1 expression after ionizing radiation (6 Gy) in A549/X cells transduced with shRNA targeting FLOT1 or control shRNA. Each experiment was performed in triplicate. Relative expression was represented by the ratio of the gray values for the target protein and internal reference. The protein fold change was calculated and annotated under the target bands. The fold-change thresholds had to be greater than 1.2 or lower than 0.8 with a P-value<0.05. The values were presented as the mean ± SD. *p < 0.05; **p < 0.01; ***p < 0.001; ****p < 0.0001.

To determine the potential binding proteins of PD-L1 and further identify the biomarkers of patients’ response to radiotherapy, IP-MS was performed to identify the potential proteins that interact with PD-L1. Given that FLOT1 was reported to facilitate tumor development by regulating protein stability, FLOT1 was selected, from 29 protein molecules detected by IP-MS (Supplementary material S1), as the subject in this study. Subsequently, the interaction of FLOT1 with PD-L1 identified in MS was further validated by Co-IP assays. As shown in Figure 1a, the interaction between FLOT1 and PD-L1 was observed in endogenous Co-IP, although the interaction was modest in parental A549 cells compared with radioresistant A549/X cells. Moreover, Flag-tagged FLOT1 was overexpressed by the presence or absence of HA-tagged PD-L1 in HEK293T cells and monitored for interaction through immunoprecipitation with anti-Flag antibody followed by immunoblotting with anti-HA antibody. We found that PD-L1 was pulled down by FLOT1 (Figure 1b), implying a protein–protein interaction between FLOT1 and PD-L1. Similarly, anti-HA immunoprecipitates from HEK293T cells transfected with HA-tagged PD-L1 and Flag-tagged FLOT1 were subjected to immunoblotting using anti-Flag antibody. As expected, the result also indicated that FLOT1 is bound with PD-L1 (Figure 1b).

To further investigate the regulatory relationship between FLOT1 and PD-L1 expression, the following experiments were performed. Our results showed that radioresistant cell lines A549/X and H520/X presented higher expression of PD-L1 and FLOT1 than parental cells (Figures 1c,d), suggesting that FLOT1 might be involved in radiation resistance. In tumor tissues from NSCLC patients, FLOT1 was dramatically upregulated compared to their matched adjacent normal lung tissues. Interestingly, the expression of PD-L1 was higher in tumor tissues with FLOT1 high relative expression than low relative expression (Figure 1e). More importantly, total cellular PD-L1 protein was dramatically reduced following transfecting shRNA targeting FLOT1 in A549/X cells (Figure 1f). Moreover, the PD-L1 protein expression was significantly upregulated at the 12th hour after radiation in A549/X cells with control shRNA, compared with A549/X cells with depletion of FLOT1 (Figure 1g). Taken together, these findings indicated that FLOT1 positively regulated the expression of PD-L1 and might be responsible for radiation resistance.

### FLOT1 knockdown alleviated radioresistance by inhibiting cell migration and impeding EMT

**Figure 2.**
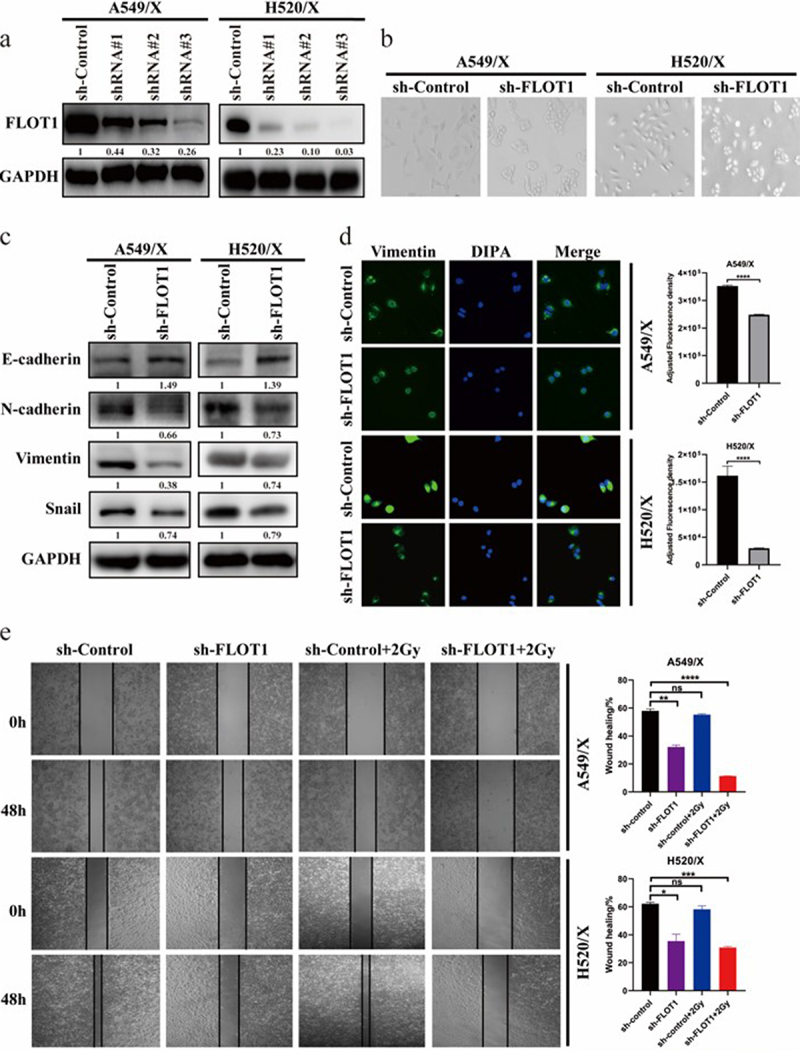
FLOT1 knockdown inhibited cell migration and impeded EMT of NSCLC cells. (a) Knockdown of FLOT1 in three specific shRNA-transduced stable radioresistant A549/X and H520/X cells. (b) Representative morphological images of the indicated NSCLC cells. (c) Western blotting analysis of EMT markers (E-cadherin, N-cadherin, Vimentin, and Snail) in the indicated cells. (d) Immunofluorescence images of Vimentin in the indicated cells. (e) Wound healing assay was performed to assess the migration of A549/X and H520/X cells with different treatments. Each experiment was performed in triplicate, and the values were presented as the mean ± SD. **p < 0.01; ****p < 0.0001; not significant (n.S); as compared with sh-Control group.

To investigate the effect of FLOT1 on radiation resistance, we prepared FLOT1 knockdown shRNAs and further transfected them to A549/X and H520/X cells by lentivirus infection, then detected the knockdown efficiency by Western blotting (Figure 2a). Cell single colony with the highest knockdown efficiency was used for subsequent studies. Interestingly, we found morphological changes from the typical spindle-like shape (mesenchymal morphology) of A549/X and H520/X cells to the cobblestone-like shape (epithelial morphology) of FLOT1 knockdown A549/X and H520/X cells (Figure 2b), suggesting that FLOT1 may endow cancer cells with EMT properties.

There is accumulating evidence that EMT closely participated in NSCLC radioresistance.^21–23^ Our previous studies also demonstrated that cells treated with radiation showed EMT,^7,8^ with concomitant upregulation of Vimentin, N-cadherin, and Snail and downregulation of E-cadherin. In this study, a typical MET phenotype, enhanced expression of epithelial marker (E-cadherin) and decreased expression of mesenchymal markers (N-cadherin, Vimentin, and Snail), was observed in FLOT1 knockdown A549/X and H520/X cells (Figure 2c). Equally, the immunofluorescence assay presented similar results of Vimentin expression (Figure 2d). These findings indicated that silencing FLOT1 might promote radiosensitivity via impeding radiation-induced EMT. Subsequently, wound healing assay was performed to examine the role of FLOT1 in radiation-induced cell migration. The results showed that radiotherapy plus silencing FLOT1 significantly inhibited cell migration capacity, and the wound healing percentage in A549/X sh-Control, sh-FLOT1, sh-Control plus radiation, and sh-FLOT1 plus radiation group was 58.00%, 32.20% (*p* = 0.0029), 55.33% (*p* =0 .0029), and 11.35% (*p* = 0.0004), respectively. The wound healing percentage in the four groups of H520/X was 62.18%, 35.56% (*p* = 0.0168), 58.26% (*p* = 0.1739), 30.90% (*p* = 0.0009), respectively (Figure 2e). Taken together, these data demonstrated that FLOT1 endowed cancer cells with EMT properties, and FLOT1 knockdown inhibited cell migration and alleviated radioresistance in NSCLC cells.

### Effect of FLOT1 on the lethality of radiation in vitro and in vivo

**Figure 3.**
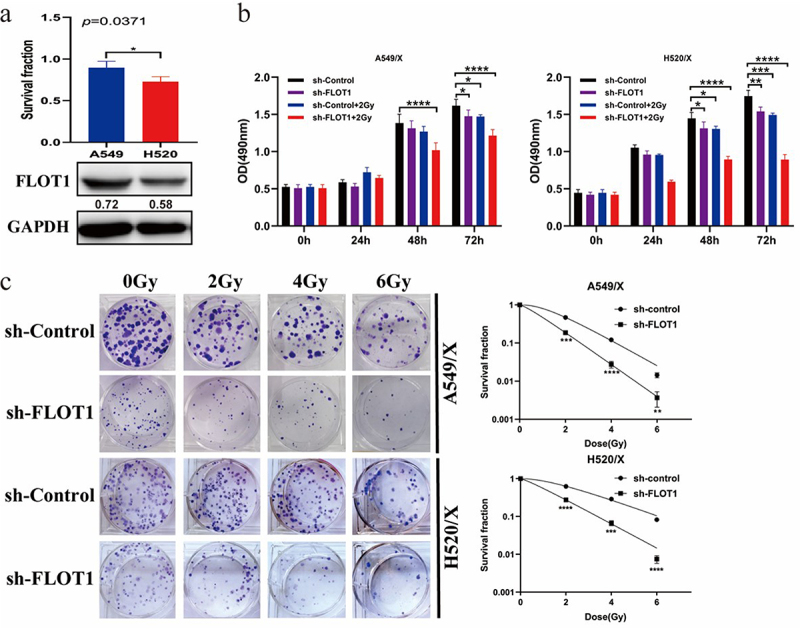
Downregulating FLOT1 increased the lethality of radiation in NSCLC cells. (a) Growth inhibition of the A549 and H520 cell lines after radiation by MTS assay, and the expression of FLOT1 in the A549 and H520 cell lines by Western blotting. (b) MTS assay was performed to assess the proliferation ability of A549/X and H520/X cells with different treatments. (c-e) A549/X and H520/X cells were exposed to increasing dose (0, 2, 4, and 6 Gy) of ionizing radiation, and the survival fractions were assessed by colony formation assay. Each experiment was performed in triplicate, and the values were presented as the mean ± SD. *p < 0.05; **p < 0.01; ***p < 0.001; ****p < 0.0001.

Next, we examined the cell survival fraction of A549 and H520 after delivery of radiation（2 Gy of radiation), and the respective survival fractions were 89% and 73% (*p* = 0.0371). We further evaluated the protein expression of FLOT1 in A549 and H520 cell lines before radiation. The data showed that FLOT1 expression in human NSCLC cell lines was positively correlated with the survival fraction, suggesting that FLOT1 might induce resistance to radiation (Figure 3a). Furthermore, the MTS assay also showed that silencing FLOT1 increased the radiation lethality in NSCLC cells (Figure 3b). Next, the colony formation assay result showed that a lower survival fraction was seen in the sh-FLOT1 group after conventionally fractionated radiotherapy, compared with the sh-Control group (Figures 3c), indicating that the depletion of FLOT1 had a synergistic effect with radiation on killing of NSCLC cells. It is noteworthy that the trend of radio-sensitization was more significant in the colony formation assay compared with the radio-sensitization in MTS assay. This could be a consequence of the longer observation time and the higher radiation dosage of the clone formation experiment. Collectively, these results suggested that FLOT1 plays a critical role in the antitumor response to radiotherapy.

**Figure 4.**
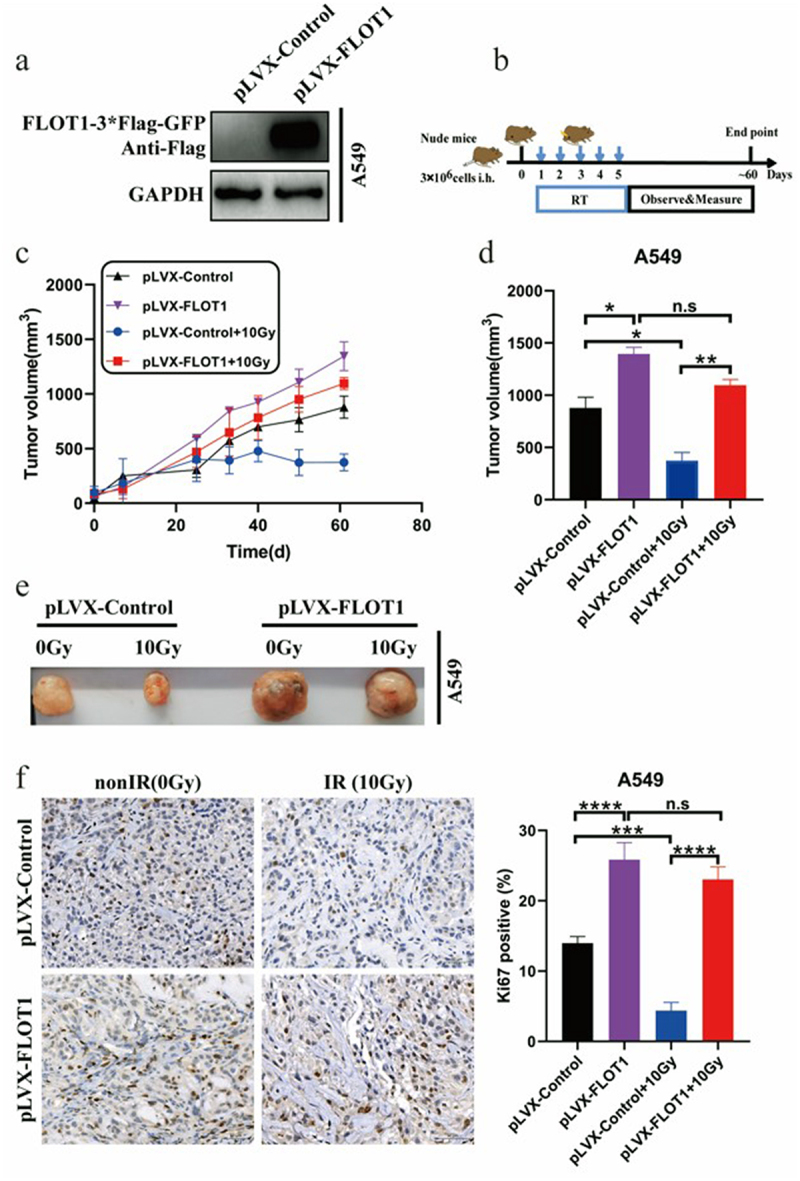
FLOT1 contributed to the growth of NSCLC cells in vivo with radiotherapy. (a) Western blotting analysis of FLOT1 expression to evaluate overexpression efficiency in A549 cells. (b) Scheme of treatment. (c) Tumor growth curves of various groups. (d) Statistical analysis of tumor volume and (e) representative images of the tumors in various groups at day 61 after local radiotherapy. (f) Representative immunohistochemical images for Ki67 protein expression in subcutaneous tumors from mice injected with A549 cells at day 61 post-radiotherapy. Scale bars, 50 μm. The data represent the mean ± SD. *p < 0.05; **p < 0.01; ***p < 0.001; ****p < 0.0001; not significant (n.S).

In the animal model, we randomly divided the nude mice into four groups: alone injected with FLOT1 overexpression A549 cells; injected with FLOT1 overexpression A549 cells plus radiotherapy; alone injected with parental A549 cells; and injected with parental A549 cells plus radiotherapy. The overexpression efficiency of FLOT1 in A549 cells was detected by Western blotting (Figure 4a). Treatment schedule and tumor growth curves are exhibited in Figures 4b,c. Comparative analyses showed that the cancerous lesion presented a larger dimension in the FLOT1 overexpression group compared to the control group. When combined with radiotherapy, the control group exhibited robust efficacy of tumor destruction, contrary to the FLOT1 overexpression group (Figure 4d,e). In other words, radiation-induced antitumor effect compromised with FLOT1 overexpression. Furthermore, the IHC analyses showed a significantly higher number of Ki67-positive cells in FLOT1 overexpression tumors compared with tumors from the control group (Figure 4f). Taken together, these results demonstrated that FLOT1 contributed to the radioresistance of NSCLC cells in vivo.

A total of 10 patients with NSCLC who received chemoradiotherapy were enrolled. Their median age was 67 years (ranged 44 to 78). In these NSCLC patients, 80% of the patients were males and all of them were never-smokers. Furthermore, FLOT1 expression was evaluated in all 10 patients. Staining of the specimens from 80% of the patients (8 of 10) was scored as FLOT1 low or none and those from 20% of the patients (2 of 10) was scored as FLOT1 high. All the 10 patients were available for response assessment after delivery of conventionally fractionated radiotherapy. Five patients achieved a partial response, three patients had stable disease, and two had progressive disease as their best response. Therefore, the overall objective response rate (ORR) was 50.0%, and the disease control rate (DCR) was 80.0%. Of note, the DCR (100% versus 0% [*p* = 0.002]) was significantly higher in patients with low or none FLOT1 expression than high FLOT1 expression (Table 1), which suggested that FLOT1 expression was inversely correlated with the response to radiotherapy in NSCLC patients.

**Table 1.** Correlations between baseline characteristics, radiotherapy response, and FLOT1 expression in patients with NSCLC.

Characteristic	Overall	FLOT1 Expression(*N* = 10)		*p* Value
		High(2)	Low/None(8)	
Age, y				0.236
≥65	8	1	7	
<65	2	1	1	
Sex				0.429
Male	8	2	6	
Female	2	0	2	
Smoking Status				-
Smoker	0	0	0	
Never smoked	10	2	8	
Histological type				0.153
ADC	4	2	2	
SQCC	4	0	4	
Other	2	0	2	
T stage				0.534
T1	3	0	3	
T2	4	1	3	
T3	2	1	1	
T4	1	0	1	
N stage				0.076
N0	4	0	4	
N1	2	1	1	
N2	3	0	3	
N3	1	1	0	
N4	0	0	0	
Stage				0.335
I	4	0	4	
II	2	1	1	
III	0	0	0	
IV	4	1	3	
PD-L1 Expression				0.766
IHC 0	4	1	3	
IHC 1	2	0	2	
IHC 2	1	0	1	
IHC 3	3	1	2	
Efficacy				
PR	5	0	5	
SD	3	0	3	
PD	2	2	0	
ORR	5	0	5	0.114
DCR	8	0	8	0.002

Abbreviations: IHC, immunohistochemistry; ADC, adenocarcinoma; SQCC, squamous cell carcinoma; PR, partial response; SD, Stable disease; PD, progressive disease; ORR, objective response rate; DCR, disease control rate.

### FLOT1 was involved in DNA damage activated STING signaling pathway and reprogrammed the tumor immune microenvironment

**Figure 5.**
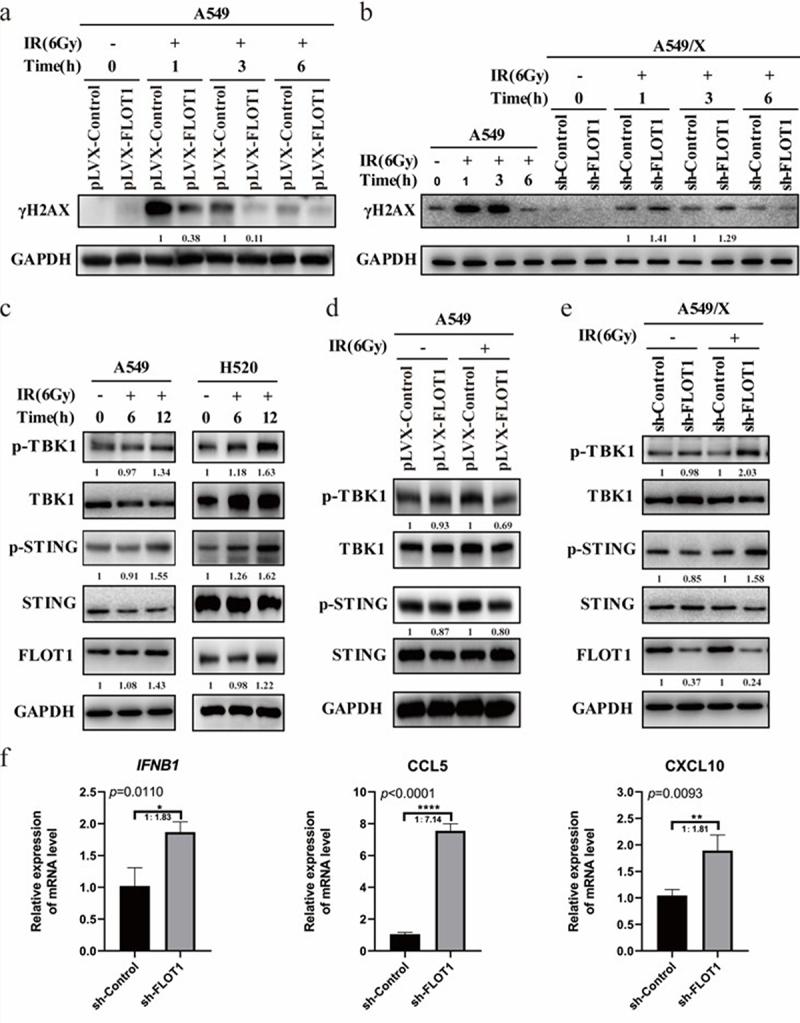
FLOT1 depletion boosted DNA damage activated STING signaling pathway. (a) Western blotting analysis of γH2A× at the indicated time points in A549 Plvx-FLOT1 and Plvx-Control group cells. (b) Western blotting analysis of γH2A× at the indicated time points in A549 cell and A549/X sh-FLOT1 and sh-Control group cells after 6 Gy radiation. (c) Western blotting analysis of FLOT1, STING, Phospho-STING, TBK1, and Phospho-TBK1 in A549 and H520 cells after 6 Gy radiation. (d) Western blotting analysis of STING, Phospho-STING, TBK1, and Phospho-TBK1 in A549 Plvx-FLOT1 and Plvx-Control groups after 6 Gy radiation. (e) Western blotting analysis of FLOT1, STING, Phospho-STING, TBK1, and Phospho-TBK1 in A549/X sh-FLOT1 and sh-Control groups after 6 Gy radiation. (f) The mRNA expression of CCL5, CXCL10, and IFNβ wasdetected by Qrt-PCR in A549/X cells with or without FLOT1 depletion after radiation. Each experiment was performed in triplicate, and the values were presented as the mean ± SD. *p < 0.05; **p < 0.01; ***p < 0.001; ****p < 0.0001.

Radiation kills cancer cells via inducing DNA damage response. As shown in Figure 5a, overexpression of FLOT1 resulted in the lower level of phosphorylated H2AX (γH2A-X) at 1 h, 3 h, and 6 h after radiation than the pLVX-Control group. In Figure 5b, A549/X cells with FLOT1 knockdown had higher level of γH2AX at 1 h and 3 h after radiation than the sh-Control group. The results indicated that FLOT1 knockdown promoted radiation-induced DNA damage, thereby inhibiting cell proliferation and enhancing the lethality of radiation. Moreover, A549/X cells presented lower γH2A-X than parental cells under the same treatment condition (Figure 5b), confirming the radioresistant characteristics of the A549/X cells.

Radiation-induced DNA damage leads to the activation of the STING pathway,^24^ which was confirmed in our study as well (Figure 5c). FLOT1 depletion boosted radiation-induced DNA damage. Hence, we sought to explore the effect of FLOT1 on STING signaling pathway. Figure 5d shows that the phosphorylation of STING and TBK1 were significantly decreased in pLVX-FLOT1 group after radiation, contrary to the pLVX-Control group. However, the inhibition of FLOT1 showed the opposite effect on STING signaling in Figure 5e. The STING pathway has been previously demonstrated to dictate the transcription of chemokine CCL5, CXCL10, and type I IFN.^20^ Our results showed that FLOT1 knockdown caused significant increase in the mRNA expression of CCL5, CXCL10, and IFNB1 in A549/X cells (Figure 5f). In short, these findings suggested that FLOT1 depletion promoted radiation-mediated activation of STING signaling pathway and FLOT1 overexpression have the opposite effect on STING signaling pathway activation.

**Figure 6.**
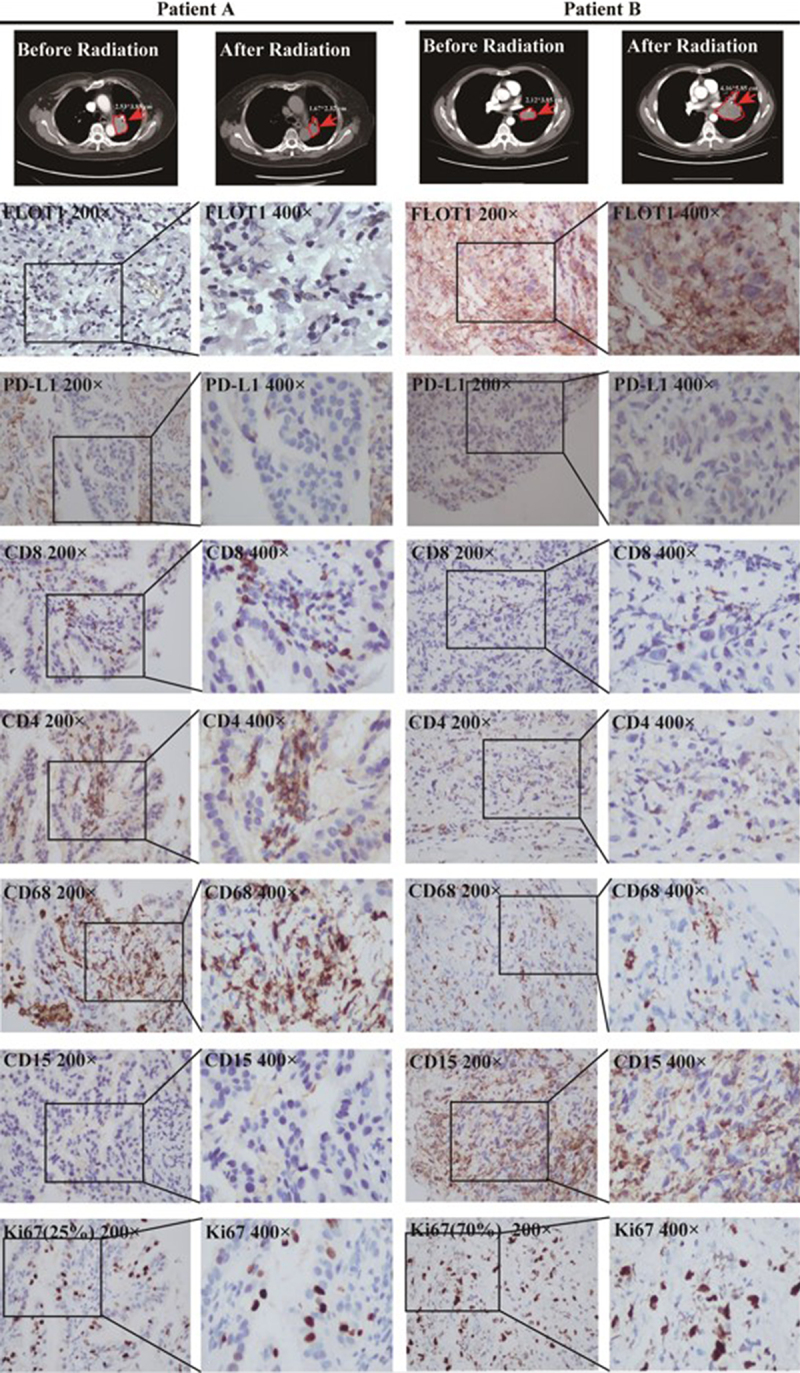
The correlation between FLOT1 and tumor immune microenvironment. The anti-CD4, anti-CD8, anti-CD68, anti-CD15, and anti-FLOT1, anti-PD-L1, anti-Ki67 antibodies were used to detect infiltration of T cells, macrophages, neutrophils and the expression of FLOT1, PD-L1, and Ki67, respectively, in two representative patients by IHC.

STING and PD-L1 signaling pathways are pivotal players in immune responses against tumors. Considering that FLOT1 is the upstream signaling molecule of STING and PD-L1 signaling pathway, we further explored whether FLOT1 was involved in the immune regulation of tumor microenvironment. Figure 6 shows that tumor foci with lower FLOT1 expression (Patient A vs. Patient B) had more infiltration of CD8+ T cells, CD4+ T cells, and CD68+ tumor-associated macrophages and less infiltration of CD15+ neutrophils, indicating that FLOT1 induced immunosuppressive microenvironment and helped tumor to circumvent immune surveillance.^7,25–28^ Subsequently, we also observed that tumor in a higher immune suppressive context had a higher Ki67 proliferation index (70% vs. 25%). Taken together, these data suggested that FLOT1 decreased antitumor immunity and enhanced radioresistance by regulating tumor microenvironment.

## Discussion

From 29 potential candidates of PD-L1 interactors identified by IP-MS analysis, we chose FLOT1, a key regulator in endocytosis and degradation of proteins and T-cell activation,^29^ to investigate its relationship with radioresistance, which has not been explored by other researchers. In this study, we demonstrated that the expression of FLOT1 is inversely correlated with response to radiotherapy in NSCLC cell lines, subcutaneous mouse xenograft tumor model, and patients. Hence, FLOT1 may be a potential biomarker to predict the response to radiotherapy. These are novel, previously undescribed functions of FLOT1 in NSCLC radiotherapy.

Mechanistically, the radioresistant cells showed less DNA damage than radioresistant cells with FLOT1 depletion after radiotherapy. PD-L1 has been reported to protect tumor cells from DNA damage after DNA-damaging therapy.^30^ We, therefore, speculated that FLOT1 was responsible for protecting DNA from damage by regulating PD-L1 expression. Moreover, FLOT1 facilitated the EMT process and stimulated cell migration in this research, which was also one of the mechanisms by which FLOT1 enhanced radioresistance.

In this study, considerable attention was focused not only on the impact of FLOT1 on tumor cells directly but also on the immune microenvironment that affects the radioresistance in NSCLC. The STING pathway is a bridge to connect innate and adaptive immunities.^31^ STING agonists have been demonstrated to elicit or augment anti-tumor immune responses in a myriad of pre-clinical studies^32^ and exhibit a synergistic effect with radiotherapy.^24^ The currently published phase I and dose-escalation clinical trials, however, reveal that the clinical activity of STING agonists is far lower than expected based on the potent antitumor activity observed in pre-clinical models. The overall response rate is merely 2.1% (1 patient with partial response in 47 patients, no complete response) in patients with single-agent MIW815 delivery.^33–35^ No complete or partial responses in MK-1454 monotherapy arm were observed (total 26 patients).^36^ In our study, FLOT1 depletion enhanced the activation of STING pathway by boosting DNA damage and increasing the accumulation of dsDNA in cytoplasm after delivery of radiation, which might compensate for the limitation of the exogenous CDN delivery – low cell permeability and unsatisfied activation of human STING.^37^ Hence, the activation of STING signaling and promotion of anti-tumor immunity through inhibiting FLOT1 might provide new insights for anti-tumor therapy in NSCLC.

Intriguingly, we observed that PD-L1 was reduced following FLOT1 depletion at the cell level; however, there was no significant association between PD-L1 and FLOT1 expression in NSCLC patients (Table 1). For example, FLOT1 expression was strongly positive on Patient B, whereas PD-L1 expression of both Patient A with immune promoting microenvironment (partial response to radiotherapy) and Patient B with immunosuppressive microenvironment (progressive disease to radiotherapy) were negative (TPS <1%) assessed by IHC. Growing evidence has demonstrated that PD-L1 expression is not an ideal biomarker to predict immune response.^38^ Consequently, it is reasonable to speculate that the mechanism of immune microenvironment regulation by FLOT1 does not strictly depend on the PD-L1 signal pathway, and FLOT1 may have a better predictive value than PD-L1 in radioimmunotherapy. In other words, the combined detection of FLOT1 and PD-L1 will be a more reliable indicator for therapy and prognosis in NSCLC.

In summary, this study is the first, to the best of our knowledge, to unveil that NSCLC cells can elevate FLOT1 expression to launch a defense against radiotherapy through stimulating cell migration, impeding radiation-induced DNA damage, and inducing tumor immune desertification. The molecular mechanisms mainly involved the activation of the STING pathway. These findings provide a novel biomarker and potential therapeutic target for reducing radioresistance in NSCLC.

## Materials and methods

### Cell culture and establishment of radioresistant cells

A549, H520, and HEK293T cells were obtained from the American Type Culture Collection (Manassas, VA). Radioresistant cells were selected by conventionally fractionated radiation in our laboratory as follows. A549 and H520 cells in the logarithmic growth phase received radiation from a TrueBeam linear accelerator (Varian Medical Systems, Palo Alto, CA, USA). The radiation field was 10 × 10 cm. The source skin distance was 100 cm. Radiation was delivered at 2 Gy per fraction once a day for a total of 25 fractions. After delivering 25 fractions, we subcultured cells every 3 days with no more than 15 passages. We screened A549 and H520 radioresistant cells and named them A549/X and H520/X cell line, respectively.

All cells were cultured in Dulbecco’s modified Eagle medium (Hyclone, Logan, UT) containing 10% fetal bovine serum (Life Technologies, Grand Island, NY) and 100 U/ml Penicillin–Streptomycin (Hyclone, Logan, UT) at 37°C with 5% CO_2_. All cell lines used for research were cultured for fewer than 20 generations and routinely screened to confirm the absence of mycoplasma contamination.

### Vectors, transfection, and retroviral infection

To silence endogenous FLOT1, three shRNA oligonucleotides were synthesized. pLVX-FLOT1 overexpressing human FLOT1 was generated by subcloning the polymerase chain reaction-amplified human FLOT1 coding sequence into a pLVX vector. The retroviral vectors mentioned above (2 μg) were transfected into HEK293T cells using the Lipofectamine 2000 reagent (Invitrogen). The media containing lentivirus was collected at 48 h and 72 h after transfection and stored at −80°C. Retroviral infection was performed using a viral supernatant supplemented with polybrene (8 μg/ml, Santa Cruz Biotechnology) by incubating the cells. After overnight incubation, the medium was aspirated and replaced with full medium. After 48 h, cells were trypsinized and plated into new 24-well plates with a culture medium containing 4 µg/ml puromycin. After 1 week of puromycin selection, cells were trypsinized and plated on 96-well plates with an indicated density of 1 to 3 cells in one well. Culture medium containing 2 µg/ml puromycin was used for continued selection. After 1 to 2 weeks, many single colonies had formed. These single colonies were initially transferred into 24-well plates and then later into 6-well plates for further expansion. Cell lysates from these single colonies were prepared and used for Western blotting to validate the FLOT1 knockdown and overexpression effects in these cells. The oligonucleotide sequences of the shRNA against FLOT1 and FLOT1 overexpression are provided in Table S1.

### Colony formation assay

Colony formation assays were performed as previously described.^7^ The survival fraction of cells was calculated as follows: survival fraction = the colony formation rate in the treatment group/control group. Survival curves were fitted by using the single hit multi-target model (*Y = 1 - (1 - exp (-k × x)) ^ N*) with GraphPad Prism version 8.0.

### MTS assay

Cells were seeded into 96-well plates at an indicated density of 1000 cells per well and were incubated in 100 µl of DMEM containing 10% FBS. For different treatment conditions described in the paper, each condition was replicated 6 times. At different time points, MTS cell proliferation assays were performed using CellTiter 96 AQueous One Solution Cell Proliferation Assays (MTS) (Promega G3580) according to the manufacturer’s instructions.

### Wound healing assay

Wound healing assays were used to evaluate cell motility induced by irradiation. Approximately 1 × 10^5^ cells were plated in 6-well plates, a wound was scratched by a 10 µl pipette tip when the cell layer reached about 90% confluence. After aspirating the separated cells, the culture medium containing 1% FBS (2 ml/well) was added to 6-well plates, and then the images were obtained by a microscope with a 4× objective at 0 h as a control. The cells were further cultured for 48 h at 37°C. Then, the wound images of the same location were photographed again by a microscope with a 4× objective. At least three fields were observed in each independent experiment. The wound area was quantified by the Image J software, and the percentage wound healing was calculated according to the following formula: *Percentage wound healing = [(Area0h – Area48h)/Area0h] × 100%*.

### Western blotting

Following the designated radiation treatment, RIPA lysis buffer containing protease inhibitors and phosphatase inhibitors was used to extract total protein from the cells at the indicated time points. Protein concentration was determined using BCA Protein Assay kit (Beyotime, China). The protocol used for Western blotting was described previously.^4^ The antibodies are summarized in Table S2.

### Real-time quantitative polymerase chain reaction (qRT-PCR)

Total RNA was extracted using RNAiso Plus (Takara, Japan) and quantified using a One DropTM OD-1000+ Spectrophotometer (One Drop, USA). Then, 100 ng of total RNA was reverse transcribed into cDNA using NovoScript® Plus All-in-One First-Strand cDNA Synthesis SuperMix (gDNA Purge) (Novoprotein, China). Subsequently, qRT-PCR was performed using TB GreenTM Premix Ex TaqTM (Tli RNaseH Plus) (Takara, Japan) with Stratagene M×3000P system (Agilent Technologies, USA). Each RNA sample was run in three independent experiments. The forward and reverse primer sequences are described in Table S3.

### Co-Immunoprecipitation (Co-IP)

For endogenous protein interaction, cells were lysed in a lysis buffer (the main components: 20 mM Tris (pH7.5), 150 mM NaCl, 1% Triton X-100, 1 mM EDTA, and 1 mM of PMSF/Cocktail) for 30 min on ice. Cellular debris was cleared by centrifugation at 12,000 rpm for 10 min at 4°C, and 30 µl of supernatants was used for immunoblotting. The remaining lysates were used to perform IP reactions, incubated with primary antibody on the rotating plate at 4°C overnight, followed by the addition of 20 μl washed protein A/G agarose and incubation for a further 3 h at 4°C. The immunocomplex was quickly washed five times with 500 μl lysis buffer and eluted in equal volume 2× loading buffer at 95°C for 10 min.

For Co-IP of exogenous protein interaction, the DNA fragment of FLOT1-3Flag or PD-L1-3 HA was cloned into the pcDNA3.1 expression plasmid. HEK293T cells were seeded in 6-well plates overnight and co-transfected with corresponding constructs as shown in each figure (Flag-tagged FLOT1 was overexpressed with the presence or absence of HA-tagged PD-L1 in HEK293T cells, or HA-tagged PD-L1 was overexpressed with the presence or absence of Flag-tagged FLOT1 in HEK293T cells). The supernatants of cell lysates were harvested 48 h post-transfection in accordance with the procedures described above. The remaining lysates were immunoprecipitated with indicated antibodies magnetic beads (anti-Flag or anti-HA) at 4°C overnight. Then, the magnetic beads were washed five times with PBST (PBS containing 0.1% Triton X-100) using magnetic stand and boiled in loading buffer containing 5% β-mercaptoethanol for 5 min. The precipitates were separated by SDS-PAGE and immunoblotted as described.

### Immunofluorescence analysis

Cells were seeded on 35 mm glass-bottom cell culture dish (NEST) and cultured overnight. Next, cells were washed three times with 1 × PBS, fixed with 4% paraformaldehyde fix solution for 10 min, washed three times with 1 × PBS, permeabilized with 0.1% Triton X-100 for 15 min, washed three times with 1 × PBS, blocked with 1% bovine serum albumin in 1 × PBS for 1 h. To analyze the role of FLOT1 in epithelial–mesenchymal transition, cells were incubated with anti-Vimentin antibody (Rabbit, 1:150, Signalway Antibody) overnight at 4°C. The secondary antibody was FITC-conjugated goat anti-rabbit IgG (Yifeixue Bio, China). Cells were restained with DAPI (Beyotime, China) and imaged using Echo revolve fluorescence microscope (Echo-lab Revolve, USA). Raw fluorescent cell images were analyzed in the Image J software. Adjusted Vimentin fluorescence intensity was calculated according to the following formula: *Adjusted fluorescence intensity = the integrated density of the selected region − (area of the selected region × mean fluorescence of background readings)*.^39,40^

### Tumor growth and treatments

3–4 weeks, 12–15 g, female, nude mice were purchased from Shanghai SLAC Laboratory Animal Co., Ltd (SLAC, China). All animal studies were approved by the Institutional Animal Care and Use Committee of Tongji University School of Medicine and were performed according to Institutional Guidelines and Protocols. First, cells (3 × 10^6^) were subcutaneously injected into the right outer thighs of the nude mice. When the tumor increased to 5–10 mm, tumor bearing mice received 2 Gy of the fractionated radiotherapy per day for 5 days.

The longest dimension (L) and shortest dimension (W) of tumor were measured three times per week with a digital caliper, and the volume of tumor was calculated according to the following formula: *Tumor Volume (mm*^*3*^*) = L×W*^*2*^*/2*. All nude mice were sacrificed after 2 months, and tumors were harvested for immunohistochemical analysis. Ki67-positive cells and cancer cells were counted, and Ki67 proliferation index (percentage of Ki67-positive cells) calculated as Ki67-positive cells/cancer cells × 100%.

### Patients and tissue collection

A total of 10 NSCLC patients who received chemoradiotherapy were enrolled in the present study. Their tumor tissues were derived from endobronchial ultrasound (EBUS) guided fine needle aspiration or CT-guided percutaneous core-needle lung biopsy before radiotherapy. All tissues were fixed in 10% neutral-buffered formalin and stored as paraffin-embedded archival (FFPE) samples. Moreover, all tissues were reviewed by experienced pathologists for confirmation of histological type and a tumor content higher than 30%. This study was approved by the Ethics Committee of Shanghai Pulmonary Hospital, Tongji University, and written informed consent was obtained from each participant before any study-related procedure.

### Immunohistochemistry (IHC)

FFPE tissue blocks, 4 μm thick, were transferred to glass slides, and IHC analyses were performed as previously described.^7,8^ The antibodies are summarized in Table S4. Notably, the degree of FLOT1 immunostaining in NSCLC was scored based on both the proportion of positively stained tumor cells and the intensity of staining. The proportion of tumor cells was scored as follows: 0 (no positive tumor cells), 1 (<10% positive tumor cells), 2 (10–50% positive tumor cells), and 3 (>50% positive tumor cells). The intensity of staining was graded according to the following criteria: 0 (no staining); 1 (weak staining = light yellow), 2 (moderate staining = yellow brown), and 3 (strong staining = brown). The staining index (SI) was calculated as the staining intensity score proportion of positive tumor cells. Specimens were scored as 0, 1, 2, 3, 4, 6, and 9 determined by the staining index. As previously identified by Shi-Hong Zhang et, al, patients with the SI score ≥ 4 were considered as high FLOT1 expression level and ≤ 3 represented low or none FLOT1 expression.^41^

### Statistical analysis

Statistical analysis was performed using GraphPad Prism version 8.0, and all experiments were repeated three times. Quantitative values were presented as mean ± standard deviation (SD). A Student’s t-test was used for comparisons between two groups. For all analyses, the *P* value less than 0.05 was considered statistically significant, and *P* values were represented as *, *p < 0.05, **p < 0.01, ***p < 0.001, ****p < 0.0001, and not significant (n.s).

## Supplementary Material

Supplemental Material

## Data Availability

The data used to support the findings of this study are included within the article. The datasets and materials in the current study are available from the corresponding author on reasonable request.

## Abbreviations

NSCLC, non-small cell lung cancer; PD-L1, programmed cell death 1 ligand 1; FLOT1, flotillin-1; sh-Control, FLOT1-knockdown control; sh-FLOT1, FLOT1 knockdown; EMT, epithelial–mesenchymal transition; γH2A×, phospho-histone H2A×; cGAS, cyclic guanosine monophosphate–adenosine monophosphate synthase; STING, stimulator of interferon genes; TBK1, TANK binding kinase 1; IFN, interferon
