# Supplementary material for: Flotillin-1 enhances radioresistance through reducing radiation-induced DNA damage and promoting immune escape via STING signaling pathway in non-small cell lung cancer
Source: Cancer Biol Ther. 2023 May 2;24(1):2203332. doi: 10.1080/15384047.2023.2203332 (PMC10158545; doi:10.1080/15384047.2023.2203332)
Supplement: Supplemental Material [file KCBT_A_2203332_SM8430.zip › Supplemental material/Supplementary Material 2.docx]

Supplementary Material 2

**Supplementary Table 1.** The oligonucleotide sequences of the shRNA against FLOT1 and overexpression FLOT1.

| **FLOT1** | **5'-3'** | **Primer sequences** |
| --- | --- | --- |
| shRNA#1 | For | GATCCGGCACGATGACCAGGACTATTTGGGTACCCAAATAGTCCTGGTCATCGTGTTTTTG |
|  | Rev | AATTCAAAAACACGATGACCAGGACTATTTGGGTACCCAAATAGTCCTGGTCATCGTGCCG |
| shRNA#2 | For | GATCCGGATAGCTGAAGTTGCCTGAATGGGTACCCATTCAGGCAACTTCAGCTATTTTTTG |
|  | Rev | AATTCAAAAAATAGCTGAAGTTGCCTGAATGGGTACCCATTCAGGCAACTTCAGCTATCCG |
| shRNA#3 | For | GATCCGGGCAGAGAAGTCCCAACTAATTGGTACCAATTAGTTGGGACTTCTCTGCTTTTTG |
|  | Rev | AATTCAAAAAGCAGAGAAGTCCCAACTAATTGGTACCAATTAGTTGGGACTTCTCTGCCCG |
| Overexpress-ion | For | GCCTCGAGGCCACCATGGACTTTTTCACTTGTGGCCCAAAT |
|  | Rev | CTTATCGTCGTCATCCTTGTAATCGGCTGTTCTCAAAGGCTTGTG |

**Supplementary Table 2.** List of antibodies used for Western blotting.

| **Antibody** | **Company** | **Cat#** | **Dilution** |
| --- | --- | --- | --- |
| FLOT1 | ProteinTech | 15571-1-AP | 1:1500 |
| PD-L1 | Abcam | ab213524 | 1:1000 |
| Flag | Sigma | F3165 | 1:2000 |
| HA | Sigma | H6908 | 1:1000 |
| γH2AX | Cell Signaling Technology | 9718S | 1:1000 |
| E-cadherin | Signalway Antibody | 21473-1 | 1:1000 |
| N-cadherin | Santa Cruz Biotechnology | Sc-7939 | 1:500 |
| Vimentin | Signalway Antibody | 21488-1 | 1:1000 |
| Snail | ABclonal | A11794 | 1:500 |
| cGAS | Cell Signaling Technology | 31659S | 1:1000 |
| STING | Cell Signaling Technology | 13647S | 1:1000 |
| Phospho-STING | Cell Signaling Technology | 50907T | 1:1000 |
| TBK1/NAK (D1B4) | ABclonal | A3458 | 1:500 |
| Phospho-TBK1/NAK | ABclonal | AP106 | 1:500 |
| GAPDH | Abcam | ab181602 | 1:10000 |
| β-actin | Signalway Antibody | 21338-1 | 1:5000 |

**Supplementary Table 3.** The primer sequences used for qRT-PCR.

| **Genes** | **5'-3'** | **Primer sequences** |
| --- | --- | --- |
| IFNB1 | For | TCTGGCACAACAGGTAGTAGGC |
|  | Rev | GAGAAGCACAACAGGAGAGCAA |
| CCL5 | For | CCAGCAGTCGTCTTTGTCAC |
|  | Rev | CTCTGGGTTGGCACACACTT |
| CXCL10 | For | GTGGCATTCAAGGAGTACCTC |
|  | Rev | TGATGGCCTTCGATTCTGGATT |
| GAPDH | For | AGGTCGGTGTGAACGGATTTG |
|  | Rev | TGTAGACCATGTAGTTGAGGTCA |

**Supplementary Table 4.** List of antibodies used for Immunohistochemistry.

| **Antibody** | **Company** | **Cat#** | **Dilution** |
| --- | --- | --- | --- |
| FLOT1 | Cell Signaling Technology | 18634T | 1:100 |
| PD-L1 | Cell Signaling Technology | 13684S | 1:100 |
| CD4 | Novus Biologicals | 19371 | 1:500 |
| CD8 | Novus Biologicals | 49045SS | 1:100 |
| CD68 | Novus Biologicals | NB100-683 | 1:100 |
| CD15 | Abcam | ab135377 | 1:50 |
| Ki 67 | Cell Signaling Technology | 9449T | 1:500 |

## Supplementary Figure 1


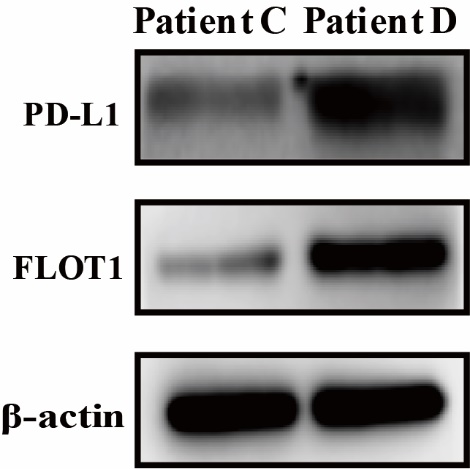


**Figure S1.** Western blotting analysis of FLOT1 and PD-L1 in Patient C and Patient D.
